# Modular Performance Analysis of a Cascaded TDM-MIMO FMCW Radar for Short-Range Counter-UAV Sensing

**DOI:** 10.3390/s26123930

**Published:** 2026-06-20

**Authors:** Dokhyl AlQahtani, Emad A. Mohamed

**Affiliations:** Department of Electrical Engineering, College of Engineering, Prince Sattam bin Abdulaziz University, Al Kharj 16278, Saudi Arabia; dm.alqahtani@psau.edu.sa

**Keywords:** Cramér–Rao bound, direction of arrival, FMCW radar, micro-Doppler, millimeter wave, MIMO radar, MUSIC, TDM-MIMO, UAV detection

## Abstract

Small unmanned aerial vehicles are difficult short-range radar targets because their millimeter-wave radar cross-sections often fall between −10 and −25 dBsm. This paper presents a modular analytical and simulation-based benchmark of a cascaded 77 GHz TDM-MIMO FMCW radar with 12 transmitters and 16 receivers, yielding a 192-element virtual ULA over a 40 m instrumented range. The framework is organized around the main counter-UAV sensing functions: range–Doppler processing first evaluates target observability and provides range–Doppler gates; Doppler-dependent TDM phase compensation is then required before virtual-array snapshots are formed for DoA estimation; and a separate long-dwell single-transmitter branch evaluates micro-Doppler separability using handcrafted features and a nearest-centroid Mahalanobis classifier. Four benchmarks are considered: detection under Swerling fluctuation models, residual TDM phase error caused by Doppler quantization, DoA estimation under an idealized far-field snapshot model, and micro-Doppler separability among UAV and bird classes. Under Swerling I, targets with a mean RCS of −10 dBsm or larger maintain detection probability above 0.9 throughout the 40 m window, whereas the −20 and −25 dBsm classes fall below that level at about 28 m and 21 m. In the far-field DoA benchmark, TLS-ESPRIT gives the lowest conditional RMSE and remains about 13–14 dB above the subarray CRLB at moderate SNR; however, these angular results are reference ceilings because the short-range operating region violates the full-aperture far-field condition and because residual TDM phase error can be severe without accurate compensation. In the micro-Doppler benchmark, birds exceed 95% per-class accuracy at 20 dB total SNR, but overall four-class accuracy saturates near 72–75% and UAV-only three-class accuracy near 63%, with most confusion between the micro-quadrotor and fixed-wing classes. This study therefore identifies architecture-specific performance margins and limitations before measured-data field validation, rather than claiming complete deployment-level performance.

## 1. Introduction

Small UAVs have become a recurring short-range security problem at airports, power facilities, prisons, border corridors, and public events. They are cheap, quick to launch, and small enough to slip under conventional radar coverage. The counter-UAV problem at short range extends well beyond bare detection: the sensor must acquire the target early, localize it precisely enough to cue a response, and separate it from birds or benign clutter before the engagement window closes. Micro-UAVs (wingspan < 1 m) and nano-UAVs (wingspan < 15 cm) aggravate all of these tasks. Composite airframes, small cross-sectional area, and motion-induced RCS fluctuation suppress observability [1,2], and millimeter-wave measurements place representative mean cross-sections between roughly −10 and −25 dBsm, depending on aspect and airframe geometry [3].

Counter-UAV systems increasingly combine radar with RF, EO/IR, and acoustic sensing because no single modality remains reliable across all engagement conditions [4,5]. Fusion offsets individual weaknesses (e.g., RF interception requires cooperative emissions, EO/IR degrades in fog, and acoustics saturate in urban noise [6]), but radar retains a central role in the short-range layer because it furnishes range, radial velocity, and angle in darkness and adverse weather without relying on the target’s own transmissions [4]. Range, velocity, and angle feed track initiation and classifier cueing; any shortfall in detection range, angular precision, or class separation directly erodes the node’s operational value. At 77 GHz, FMCW sensors are especially suited to compact counter-UAV nodes. A 960 MHz chirp bandwidth delivers sub-decimeter range resolution, and the roughly 4 mm wavelength permits densely packed arrays and narrow beams in a modest form factor [7]. These attributes matter the most at a 10–40 m standoff, where multiple objects may populate adjacent range cells with similar radial velocities and where hardware footprint constrains distributed deployment. Recent work confirms that range–Doppler representations from such radars can feed learning-based drone detection and classification pipelines when the received signal quality is adequate [8,9]. Millimeter-wave operation does not eliminate the core difficulty: small drones remain close to the thermal noise floor, and the short ranges typical of perimeter defense can place targets inside the near field of a large synthetic aperture.

MIMO radar offers a direct path to finer angular discrimination without a physically continuous array. A co-located TDM-MIMO architecture synthesizes a virtual aperture whose element count equals the product of the transmit and receive channel counts [10,11]. Cascading multiple transceiver ICs on a single board extends the achievable MIMO aperture beyond the channel budget of any one chip, a topology now standard across many millimeter-wave platforms [12]. Li et al. [13] provide a thorough tutorial on the DFT-based processing chain for such sensors (range-Doppler map formation, Doppler-induced phase correction, and virtual-array assembly). A large virtual aperture is attractive for counter-UAV work because it can resolve targets that overlap in range–Doppler space yet subtend a small angular difference. Block-TDM shrinks the effective unambiguous velocity referenced to the full MIMO cycle, plants motion-induced phase offsets across transmit blocks, and invalidates naive far-field steering when targets sit close to the array [14,15]. Each of these effects limits the detection, localization, and classification performance achievable by a cascaded front end in a short-range defensive role.

The published literature addresses these capabilities mostly in isolation. Measurement campaigns by Ritchie et al. [16], de Wit et al. [17], and Semkin et al. [3], together with the review of Coluccia et al. [18] and the broad survey of Khawaja et al. [4], pin down realistic RCS and micro-Doppler envelopes for small drones and birds. On classification, the micro-Doppler framework of Chen [19] remains a reference point, and Molchanov et al. [20] demonstrated that handcrafted spectral features can separate birds from UAVs effectively. More recent work pushes toward periodic-motion descriptors and deep classifiers: Duan et al. [21] exploited Doppler-spectrum periodicity with recurrent networks; Park and Park [9] paired low-uncertainty spectrogram images with ultra-lightweight CNNs; Mandal et al. [22] classified flying objects from UAV-borne radar; Wu and Liu [23] fused two-stream micro-Doppler representations for few-shot aerial-target recognition. Lightweight CNNs running directly on range–Doppler maps have been shown to slash computational cost while preserving competitive accuracy [24], and comparative benchmarks on 60 GHz data reveal that noise robustness is tightly coupled to network topology [25]. A recent experiment by Kozlov et al. [26] showed that even modest artificial modulation of a drone’s blade returns can camouflage it as a helicopter, underscoring the adversarial fragility of signature-based classifiers. On angle estimation, MUSIC [27], ESPRIT [28], forward–backward spatial smoothing [29], and MDL model-order selection [30] remain the core toolbox. Extensions to beamspace processing with joint phase-error correction [31], spatial interference suppression [32], iterative adaptive estimation [33], and deep-unfolded solvers [34] have been explored for millimeter-wave arrays with limited snapshots or imperfect array models.

Across the cited studies, detection, angle estimation, and micro-Doppler classification are usually reported separately rather than under one fixed cascaded TDM-MIMO configuration. Detection range is governed largely by RCS fluctuation and link margin. Angular accuracy hinges on aperture extent, phase integrity across TDM blocks, and steering-model fidelity. Micro-Doppler classification depends not only on the SNR but also on whether the chosen feature representation retains the motion cues that separate birds from UAVs and one UAV class from another. When these capabilities are evaluated on unrelated hardware in separate publications, it is unclear whether a reported limitation originates in the algorithm or in the radar architecture.

**Figure 1:**
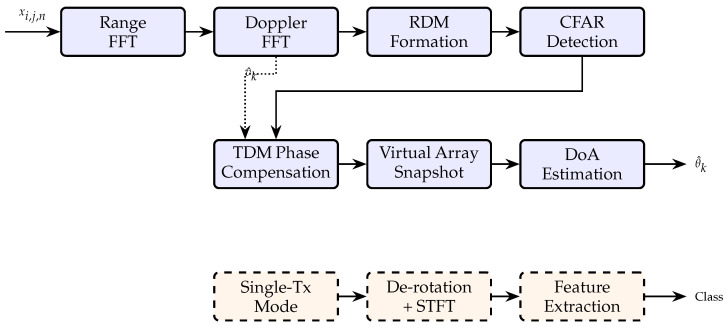
Schematic processing workflow of the cascaded TDM-MIMO FMCW radar for short-range counter-UAV sensing. Solid: MIMO flow; dashed: single-Tx micro-Doppler mode. Each branch is benchmarked under its own assumptions.

In this paper, a fixed 12Tx × 16Rx, 77 GHz cascaded configuration, yielding a 192-element virtual ULA, is assessed through four separate but connected benchmarks: detection under Swerling fluctuation models, residual TDM phase error from Doppler quantization, DoA estimation in an idealized far-field snapshot setting, and micro-Doppler separability among UAV and bird classes using interpretable handcrafted features and a low-capacity classifier. The contribution is therefore not a new detector, DoA estimator, or classifier in isolation. Rather, the contribution is a modular system-level benchmark that places these established tools under one common cascaded TDM-MIMO radar parameterization and shows where the main performance margins and bottlenecks occur. The relationship between the resulting benchmark and representative prior studies is discussed after the results are presented. The scope of the work is also important. The present study is analytical and simulation-based; it is not intended to serve as a complete experimental validation of a deployable counter-UAV system. The simulation framework deliberately isolates the detection, angular estimation, TDM phase-error, and micro-Doppler modules so that the effect of each assumption can be interpreted clearly. Consequently, the results should be read as feasibility and upper-bound reference results under controlled assumptions. Real deployment requires additional validation with measured 77 GHz drone and bird data and must account for clutter, multipath, inter-chip calibration residuals, mutual coupling, target aspect variation, near-field propagation, and imperfect TDM phase compensation. Figure 1 summarizes this cascaded TDM-MIMO FMCW radar workflow, linking the MIMO range–Doppler detection and DoA branch with the separate single-transmitter micro-Doppler branch for short-range counter-UAV sensing.

The remainder of this paper proceeds as follows: Section 2 defines the radar configuration and SNR conventions, Section 3 describes the processing chain, Section 4 presents the benchmarks, practical limitation discussion, and comparison with representative prior studies, and Section 5 concludes the paper.

## 2. System Model

Because each benchmark module uses a different observation model, the SNR conventions must be distinguished at the outset. Table 1 summarizes the three definitions: detection uses the post-combining SNR in decibels, the DoA benchmark a per-source per-element snapshot SNR, and the micro-Doppler study the total signal power (body plus blade returns) relative to the noise floor.

### 2.1. Radar Architecture and Signal Model

A monostatic cascaded TDM-MIMO FMCW front end at carrier frequency $f_{c}$ cycles $N_{\mathrm{Tx}}$ transmitters through $N_{\mathrm{Rx}}$ receivers, acquiring virtual-array measurements sequentially. Commercial realizations mount four transceiver ICs on a single board (three transmitters and four receivers per device), giving $N_{\mathrm{Tx}}=12$ and $N_{\mathrm{Rx}}=16$ in aggregate [12,13]. Inter-chip synchronization jitter and calibration residuals are set aside; ideal clock distribution is assumed throughout.

For a far-field target at angle θ, the two-way phase is

(1) $$\phi_{ij}\left(\theta \right)=\frac{2\pi }{\lambda }\left(p_{\mathrm{Tx}}^{\left(i\right)}+p_{\mathrm{Rx}}^{\left(j\right)}\right)\sin\theta ,$$

defining the virtual-array manifold [10]. Here $p_{\mathrm{Tx}}^{\left(i\right)}$ and $p_{\mathrm{Rx}}^{\left(j\right)}$ are the physical transmitter and receiver coordinates along the array axis; their sum fixes the position of the corresponding virtual element. Setting $d_{\mathrm{Tx}}=N_{\mathrm{Rx}}d_{\mathrm{Rx}}$ with $d_{\mathrm{Rx}}=\lambda /2$ produces a filled virtual ULA of $N_{v}=N_{\mathrm{Tx}}N_{\mathrm{Rx}}$ elements. For the 12Tx × 16Rx configuration this gives $N_{v}=192$ virtual elements spanning 191×λ/2≈37.2 cm at 77 GHz, corresponding to a Rayleigh beamwidth of roughly 0.53°. An aperture of this extent warrants high-resolution subspace methods alongside conventional beamforming.

Under block-TDM, the frame time is $T_{\mathrm{frame}}=N_{\mathrm{Tx}}N_{c}T_{\mathrm{PRI}}$. Doppler accumulates within each transmit block over $N_{c}$ chirps; the full MIMO frame is assembled across all $N_{\mathrm{Tx}}$ blocks. The maximum unambiguous velocity per block is $v_{\max,\mathrm{Tx}}=\lambda /\left(4T_{\mathrm{PRI}}\right)$, and the maximum instrumented range is $R_{\max}=cf_{s}/\left(2S\right)$. For the numerical configuration evaluated in this study, this corresponds to a 40 m sampled beat-frequency span. Here, “instrumented range” refers to the span of the sampled beat-frequency axis, not to guaranteed detection range for any target class. The actual detection reach is set later by the link budget and the assumed RCS fluctuation model.

Each transmitter radiates a chirp $s_{i}\left(t\right)=\exp\left(j2\pi \left[f_{c}t+S{\left(t-t_{i,n}\right)}^{2}/2\right]\right)$, with $S=B/T_{c}$. Here *t* is fast time within a chirp and $t_{i,n}$ marks the start of the *n*th chirp from the *i*th Tx channel. Dechirping maps round-trip delay to beat frequency and pulse-to-pulse phase progression to Doppler. In this standard linear-FMCW framework, range resolution is $\delta_{R}=c/\left(2B\right)$ and velocity resolution is $\delta_{v}=\lambda /\left(2N_{c}T_{\mathrm{PRI}}\right)$. Zero-padding by a factor $Z_{D}$ refines the Doppler-bin spacing to $\delta_{v}/Z_{D}$, aiding peak localization and TDM phase compensation but leaving the underlying spectral resolution, set by the coherent dwell, unchanged.

For *K* point targets, the dechirped signal at receiver *j* is

(2) $$x_{i,j,n}\left(t\right)=\sum_{k=1}^{K}\alpha_{k}\;e^{j(\phi_{k,\mathrm{beat}}+\phi_{k,\mathrm{Dop}}+\phi_{k,\mathrm{Tx}}+\phi_{k,\mathrm{Rx}})}+w_{i,j,n}\left(t\right),$$

where $\phi_{k,\mathrm{beat}}=2\pi \left(2SR_{k}/c\right)\;t$, $\phi_{k,\mathrm{Dop}}=\left(4\pi v_{k}/\lambda \right)\;t_{i,n}$, $\phi_{k,\mathrm{Tx}}=\left(2\pi /\lambda \right)\;p_{\mathrm{Tx}}^{\left(i\right)}\sin\theta_{k}$, and $\phi_{k,\mathrm{Rx}}=\left(2\pi /\lambda \right)\;p_{\mathrm{Rx}}^{\left(j\right)}\sin\theta_{k}$.

Equation (2) decomposes the received signal into range, radial-motion, and array-geometry contributions. The complex coefficient $\alpha_{k}$ absorbs the target reflectivity and any phase constant over a single coherent processing interval. The beat term pins down range; the slow-time Doppler term governs velocity estimation and TDM phase error; and the transmit/receive spatial terms encode the angle information exploited during virtual-array processing. The additive noise $w_{i,j,n}\left(t\right)$ is modeled as complex white Gaussian. Calibration mismatch, mutual coupling, clutter, interference, and hardware synchronization residuals are omitted deliberately in the main benchmark so that each processing stage can be assessed in isolation. This assumption makes the benchmark reproducible and interpretable, but it also means that the reported values should not be interpreted as guaranteed field performance. In practical deployment, these omitted effects would appear as additional model mismatch or disturbance power and would generally reduce detection range, angular accuracy, and classification reliability.

### 2.2. Link Budget, Target RCS, and Fluctuation Models

A noise-limited detection benchmark is adopted, using the monostatic radar equation to convert representative RCS values into post-integration channel SNR. After coherent integration over $N_{c}$ chirps of a transmit block, the per-channel SNR in linear scale is

(3) $${\mathrm{SNR}}_{\mathrm{ch}}^{\left(\mathrm{lin}\right)}=\frac{P_{t}\;G_{e}^{2}\;\lambda^{2}\;\sigma \;N_{c}}{{\left(4\pi \right)}^{3}\;R^{4}\;k_{B}T_{0}B_{n}\;F\;L_{\mathrm{sys}}}.$$

Converting into decibels and appending the non-coherent combining gain $G_{\mathrm{NC},\mathrm{dB}}\approx 10\log_{10}\left(\sqrt{M}\right)$ [35] for $M=N_{\mathrm{Tx}}N_{\mathrm{Rx}}$ channels yield

(4) $${\mathrm{SNR}}_{\det,\mathrm{dB}}\left(R\right)\approx {\mathrm{SNR}}_{\mathrm{ch},\mathrm{dB}}\left(R\right)+G_{\mathrm{NC},\mathrm{dB}}.$$

Equation (4) serves as a link-budget guide, not as the detector statistic. The detection threshold is calibrated via Monte Carlo simulation, imposing the false-alarm constraint directly on the range–Doppler test statistic.

Target RCS fluctuation is captured by two Swerling models. Under Swerling I [35], $f_{\sigma }\left(\sigma \right)={\bar{\sigma }}^{-1}\exp\left(-\sigma /\bar{\sigma }\right)$; under Swerling III, $f_{\sigma }\left(\sigma \right)=4\sigma {\bar{\sigma }}^{-2}\exp\left(-2\sigma /\bar{\sigma }\right)$. The Swerling I cross-section is drawn once per scan from an exponential, a model appropriate when many comparable scatterers decorrelate between dwells, as with a small multirotor shifting aspect. Swerling III adds a dominant scatterer, yielding a chi-squared density with four degrees of freedom whose mass pulls away from zero RCS. Because Swerling I places more probability near σ=0, it requires a higher mean SNR for a given $P_{d}$ and is therefore the more pessimistic model.

## 3. Integrated Signal Processing and Micro-Doppler Analysis

Figure 1 shows two processing branches that are evaluated separately. The upper branch carries the multi-channel FMCW data through range–Doppler detection and, following TDM phase compensation, assembles virtual-array snapshots for angle estimation. The lower branch draws on the same physical principles but operates in a dedicated single-transmitter mode, capturing a longer uninterrupted slow-time record for micro-Doppler characterization.

### 3.1. MIMO Branch: Detection and Snapshot Formation

Fast-time and slow-time processing form the common front end for all downstream modules. A Hann window is applied along fast time in each Tx–Rx channel before a range FFT, suppressing sidelobe leakage while concentrating beat-frequency energy. A second Hann window spans the $N_{c}$ chirps of a transmit block, followed by a Doppler FFT, yielding per-channel range–Doppler maps ${\tilde{X}}_{i,j}\left(r,d\right)$. Processing is performed one transmit block at a time because Doppler estimation at this stage uses only chirps from a common transmitter, so the detector does not require cross-block phase alignment.

Detection is based on non-coherent magnitude summation,

$$\mathrm{RDM}\left(r,d\right)=\sum_{i,j}\left|{\tilde{X}}_{i,j}\left(r,d\right)\right|,$$

with a Gaussian-approximated threshold $T_{\det}=\hat{\mu }+k_{\mathrm{fa}}\hat{\sigma }$ calibrated from $10^{6}$ noise-only realizations. Non-coherent accumulation decouples the detection stage from TDM phase integrity, avoiding the need for cross-block phase alignment before thresholding. A fixed threshold calibrated from Monte Carlo noise realizations keeps the benchmark reproducible and avoids introducing CFAR design choices as additional variables. Operational radars would typically employ CA-CFAR or OS-CFAR in cluttered environments [12]; the present study targets controlled sensitivity rather than field-tuned clutter rejection.

In the counter-UAV workflow, detection fulfills two functions. It establishes whether a low-RCS target is observable at all under the assumed fluctuation model, and it provides the range–Doppler gate from which angle estimation proceeds. The converse limitation follows directly: when two objects occupy the same or adjacent range-Doppler cells, the detector responds to their combined energy without separating them. Resolving that ambiguity is the job of the angle-processing branch benchmarked later.

Block-TDM injects a Doppler-dependent phase offset because virtual-array channels are measured sequentially. For a target at radial velocity $v_{k}$, the extra phase on the *i*th transmit block is $\Delta \phi_{i}=\left(4\pi v_{k}/\lambda \right)\left(i-1\right)N_{c}T_{\mathrm{PRI}}$; compensation relies on the intra-block velocity estimate. The worst-case residual after compensation is

(5) $$\delta \phi_{i}^{\max}=\frac{\pi (i-1)}{Z_{D}}.$$

With $N_{\mathrm{Tx}}=12$ and $Z_{D}=2$, the outermost block can accumulate up to 11π/2≈17.3 rad of residual error.

For a detected target in range–Doppler cell $\left({\hat{r}}_{k},{\hat{d}}_{k}\right)$, nominal compensation uses the Doppler-derived velocity estimate ${\hat{v}}_{k}$ before virtual-array snapshot formation:

(6) $${\tilde{X}}_{i,j}^{c}\left({\hat{r}}_{k},{\hat{d}}_{k}\right)={\tilde{X}}_{i,j}\left({\hat{r}}_{k},{\hat{d}}_{k}\right)\exp\left[-j\frac{4\pi {\hat{v}}_{k}}{\lambda }\left(i-1\right)N_{c}T_{\mathrm{PRI}}\right].$$

If the velocity estimate has error $v_{e}=v_{k}-{\hat{v}}_{k}$, the residual phase is

(7) $$\epsilon_{i}\left(v_{e}\right)=\frac{4\pi v_{e}}{\lambda }\left(i-1\right)N_{c}T_{\mathrm{PRI}}.$$

This residual produces a structured phase distortion across the virtual array. Because it grows linearly with the transmit-block index, it approximates a linear phase ramp across the Tx-major aperture, which for a ULA is equivalent to a steering offset. Its dominant first-order effect is therefore an angular bias

(8) $$\Delta \theta_{\mathrm{TDM}}\approx \frac{4v_{e}N_{c}T_{\mathrm{PRI}}}{\lambda N_{\mathrm{Rx}}\cos\theta },\;{\left|\Delta \theta_{\mathrm{TDM}}\right|}_{\max}\approx \frac{1}{Z_{D}N_{\mathrm{Rx}}\cos\theta }.$$

With $Z_{D}=2$ and $N_{\mathrm{Rx}}=16$, this gives about 1.8° at boresight, which is more than three times the nominal 0.53° Rayleigh beamwidth. Therefore, bin-quantized TDM compensation would dominate the angular error budget, and the DoA results are reported as ideal phase-compensated reference ceilings rather than deployment predictions. Practical operation would require sub-bin Doppler interpolation, phase tracking, calibration, or joint velocity–angle estimation.

After compensation, the multi-channel data for each detected target are stacked into the virtual-array snapshot x=A(θ)α+n, with $n∼\mathrm{CN}(0,\sigma_{n}^{2}I)$.

### 3.2. DoA Estimation and CRLB

Three estimators are compared, spanning a useful range of complexity and resolution capability. CBF serves as a low-complexity reference whose behavior tracks the effective aperture and window taper. MUSIC with forward–backward spatial smoothing (FBSS; $L=128\approx 2N_{v}/3$, P=65 subarrays) is the standard subspace alternative for coherent or closely spaced sources. TLS-ESPRIT exploits the shift-invariance structure of a ULA and bypasses an explicit spectral search once the signal subspace is available.

The number of sources is treated as known; in practice MDL [30] can supply an estimate. Recent millimeter-wave studies have examined beamspace formulations [31], spatial interference mitigation [32], iterative adaptive estimators [33], and deep-unfolded alternatives [34]. Such methods target scarce-snapshot or imperfect-model scenarios. A tighter benchmark is more informative here because it isolates how much angular performance the nominal virtual aperture can deliver before model mismatch becomes the limiting factor.

Steering vectors assume plane-wave propagation, noise is white Gaussian, TDM compensation is perfect, and calibration errors are absent. The Fraunhofer distance is approximately 71 m for the full aperture and roughly 31 m for the L=128 subarray, while the target ranges of interest span 10–40 m. The DoA results are therefore idealized far-field benchmarks, not direct end-to-end predictions for the actual short-range engagement geometry.

To quantify how far the short-range geometry departs from the assumed plane-wave model, consider the residual phase between the true spherical wavefront and the planar manifold. After removing the linear far-field term, the quadratic phase error is the largest at the aperture edge (x=D/2) and can be approximated as

(9) $$\delta \phi_{\mathrm{NF}}^{\max}\left(R,\theta \right)\approx \frac{2\pi }{\lambda }\frac{{\left(D/2\right)}^{2}}{2R}\cos^{2}\theta =\frac{\pi D^{2}\cos^{2}\theta }{4\lambda R},$$

where *D* is the effective aperture length. For boresight, this expression reduces to the standard far-field phase-error threshold π/8 at the Fraunhofer distance $R=2D^{2}/\lambda$. For the full $N_{v}=192$ virtual aperture, D≈0.372 m. At θ≈13°, the corresponding edge phase error is approximately 0.84π, 0.42π, and 0.21π rad, i.e., about 152°, 76°, and 38°, at R=10, 20, and 40 m, respectively. For the L=128 FBSS subarray used by MUSIC and in the CRLB, $D_{L}\approx 0.247$ m, and the corresponding values are approximately 0.37π, 0.19π, and 0.09π rad, i.e., about 67°, 34°, and 17°, at the same ranges. These values show that the plane-wave steering mismatch is non-negligible over the short-range window, especially for the full virtual aperture and for the subarray at ranges below its Fraunhofer distance. In a TDM-MIMO virtual array, the exact residual curvature depends on the physical Tx/Rx coordinates, but the aperture-level estimate above is sufficient to show that the far-field steering model is optimistic in the 10–40 m region. Such uncompensated wavefront curvature can bias MUSIC and TLS-ESPRIT estimates, broaden angular spectra, and increase the angular separation required for reliable resolution. Therefore, the angular RMSE and resolution probabilities reported below should be interpreted as far-field reference ceilings for the nominal aperture, not as direct short-range deployment predictions. A deployment-oriented DoA processor would require spherical-wave steering, near-field focusing, or range-dependent calibration.

The stochastic CRLB [36] is evaluated with $N_{\mathrm{eff}}=P$. Counting overlapping subarrays as independent makes the bound optimistic. For a single source [37],

(10) $$\mathrm{CRLB}\left(\theta \right)=\frac{6}{{\left(2\pi d_{v}\cos\theta /\lambda \right)}^{2}N_{\mathrm{eff}}L\left(L^{2}-1\right)\;\mathrm{SNR}}.$$

### 3.3. Micro-Doppler Branch: Signal Model and Classification

Micro-Doppler analysis occupies a separate branch because extracting reliable signatures demands a far longer contiguous slow-time record than the $N_{c}=128$ chirps of a single TDM transmit block. For a rotor with $N_{b}$ blades of length $L_{b}$ spinning at $f_{\mathrm{rot}}$, the micro-Doppler frequency of the *q*th blade tip is [19]

(11) $$f_{d,\mathrm{micro}}^{\left(q\right)}\left(t\right)=\frac{4\pi f_{\mathrm{rot}}L_{b}}{\lambda }\sin\;\left(2\pi f_{\mathrm{rot}}t+\frac{2\pi (q-1)}{N_{b}}\right).$$

The composite slow-time signal is $s\left(t\right)=\alpha_{\mathrm{body}}\;e^{j2\pi f_{d,0}t}+\sum_{r,q}\alpha_{b}^{\left(r,q\right)}\;e^{j\Phi_{r,q}\left(t\right)}$, with total blade power set 20 dB below the body [16,20]. The body term captures the dominant translational return; the blade terms generate the oscillatory sidebands that carry class-discriminative information. Kozlov et al. [26] recently confirmed that even modest artificial modulation of blade returns can fool trained classifiers, highlighting how fragile micro-Doppler-based identification becomes when sideband energy is marginal.

A dedicated single-transmitter mode captures an extended slow-time sequence independently of the block-TDM MIMO acquisition. The observation window is

(12) $$T_{\mathrm{obs}}=N_{\mathrm{md}}T_{\mathrm{PRI}}=0.5\;s,$$

where $N_{\mathrm{md}}=\mathrm{round}\left(T_{\mathrm{obs}}/T_{\mathrm{PRI}}\right)=7143$ consecutive chirps at $T_{\mathrm{PRI}}=70$ μs. This dwell is decoupled from the $N_{c}=128$ chirps per transmit block used in detection mode; it is a standalone classification dwell. Dispensing with inter-transmitter staggering yields a contiguous slow-time record that suits time–frequency analysis and periodicity estimation. Table 2 catalogues the class parameters and the modulation cycles captured in one $T_{\mathrm{obs}}$.

Each micro-Doppler sample is a synthetic slow-time realization of $N_{\mathrm{md}}=7143$ pulses ($T_{\mathrm{obs}}=0.5$ s). The class-level parameters ($N_{r}$, $N_{b}$, $L_{b}$, and $f_{\mathrm{rot}}$) listed in Table 2 are held constant within a class; inter-sample variability enters through two mechanisms:

- A uniformly distributed ±2 dB perturbation is applied to each rotor’s blade amplitude relative to the nominal per-blade value.
- Each rotor receives an independent initial phase drawn uniformly from [0,2π).

Independent additive white Gaussian noise is generated per sample and scaled so that the total signal power (body plus blades) meets the target SNR.

The body amplitude $\alpha_{\mathrm{body}}$ is set to an arbitrary reference level; total blade power is −20 dB relative to the body. Aspect-angle variation is excluded: all rotors and wings are observed broadside. Because class parameters are fixed and only noise, initial phase, and per-rotor amplitude vary across samples, the resulting accuracies are better understood as feature-space upper bounds than as field-deployment estimates. This is a favorable geometry: if β denotes departure from broadside, the observed micro-Doppler bandwidth scales approximately as cosβ. Thus, a 45° aspect change reduces the bandwidth feature by about 30%, which is larger than the nominal 25% tip-speed separation between the micro-quadrotor and fixed-wing classes (12 versus 16 m/s). Aspect variation, maneuvering, and environmental effects are therefore expected to increase overlap among the class feature distributions.

Time–frequency analysis uses the STFT with a Hann window of $N_{w}=128$ samples and hop size $N_{\mathrm{hop}}=4$ (96.9% overlap). With $N_{\mathrm{md}}=7143$ pulses, this produces

(13) $$N_{\mathrm{frames}}=\left\lfloor \frac{N_{\mathrm{md}}-N_{w}}{N_{\mathrm{hop}}}\right\rfloor +1=1754.$$

STFT frames spanning roughly 490 ms. The heavy overlap stabilizes the instantaneous-bandwidth sequence used in periodicity estimation while preserving the fine slow-time sampling of the spectrogram.

Body Doppler de-rotation proceeds by estimating the dominant frequency from the full-length FFT and multiplying by $e^{-j2\pi {\hat{f}}_{d,0}t}$. A DC notch (±3 bins) then suppresses the residual body peak. Without these two steps, the weaker micro-Doppler structure would be partially buried under the bulk translational line.

Four features are extracted:

1. $B_{\mathrm{md}}$ (maximum bandwidth): Spectral support width at −10 dB below the peak of the time-averaged Doppler spectrum. This feature primarily reflects the maximum radial excursion of the rotating appendages.
2. $f_{0}$ (fundamental modulation frequency): Dominant periodicity in the autocorrelation of the instantaneous-bandwidth time series (1754 frames), searched over modulation frequencies in [5,500] Hz with a minimum autocorrelation peak prominence of 0.05 and a height of 0.1.
3. $N_{h}$ (harmonic count): Peaks in the time-averaged spectrum exceeding −20 dB with ≥3 dB prominence.
4. *H* (spectral entropy): $H=-\sum_{k}p_{k}\log_{2}p_{k}$.

A no-structure gate fires when the post-notch peak-to-median ratio drops below 6 dB, returning defaults ($B_{\mathrm{md}}=0$, $f_{0}=0$, $N_{h}=0$, $H=H_{\max}$).

Classification uses a nearest-centroid Mahalanobis classifier whose pooled covariance is regularized with $10^{-6}I$. This deliberately simple baseline ensures that the reported accuracies reflect the discriminative power of the features, not the classifier’s capacity. Lightweight CNNs, image-based micro-Doppler representations, and Transformer-style models on millimeter-wave data have been shown to outperform simple baselines in noisy settings [9,24,25]. Here the objective is different: to quantify how much class separation a compact, interpretable feature space already provides, before learned representations are introduced. A direct empirical comparison with CNN- or Transformer-based classifiers is therefore deferred: a fair comparison would require measured or domain-randomized spectrogram data with aspect, motion, and environmental variability and is identified as future work rather than claimed from the present fixed-parameter synthetic dataset.

## 4. Simulation Results and Discussion

All simulations were implemented in MATLAB R2025a. System parameters appear in Table 3, the reference link budget in Table 4, and representative target RCS values in Table 5. The false-alarm probability is fixed at $P_{\mathrm{fa}}=10^{-6}$.

**Figure 2:**
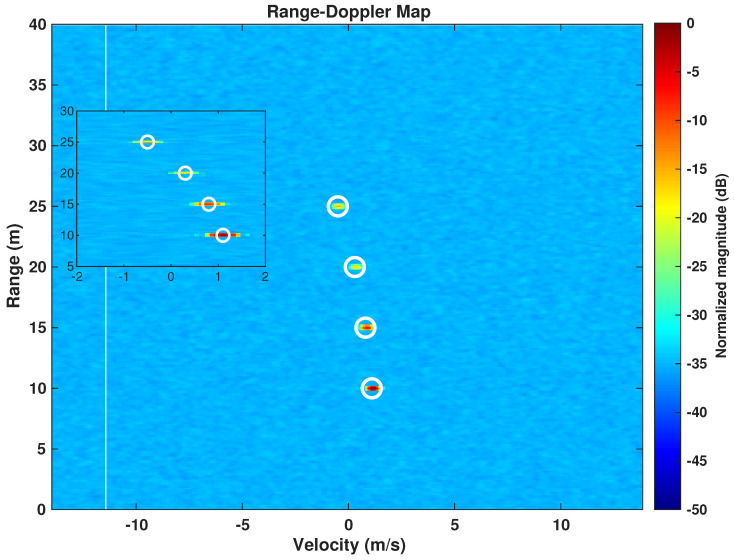
Range–Doppler map for Table 6. White circles mark the true target locations. The inset shows a zoomed low-velocity region.

Table 6 specifies a mixed scene with both isolated and co-located targets; Figure 2 shows the resulting RDM. Targets 2 and 3 share the same range–Doppler neighborhood despite a 12 dB RCS difference. Non-coherent magnitude accumulation lets the stronger micro-UAV dominate the cell, effectively masking the weaker nano-UAV. A detection in that cell does not imply that overlapping targets are separable in range–velocity space; within the present model, angle processing is the only remaining discriminant once two objects collapse onto the same coordinates. The measured SNR for Target 1 agrees with the analytical link budget to within 0.3 dB, confirming internal consistency.

### 4.1. Detection, Range, and Far-Field DoA Benchmarking

**Figure 3:**
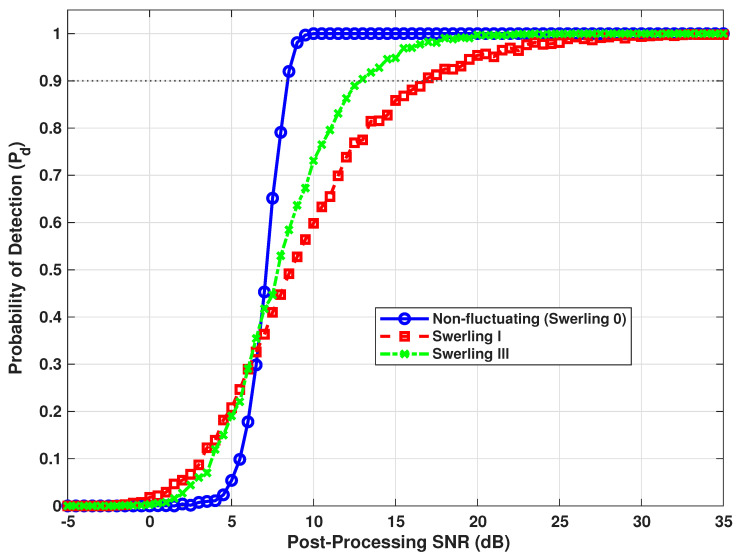
$P_{d}$ versus post-processing SNR ($P_{\mathrm{fa}}=10^{-6}$, 2000 trials).

Figure 3 quantifies the detection cost of target fluctuation at $P_{\mathrm{fa}}=10^{-6}$ (2000 trials per point). Without fluctuation, $P_{d}\approx 0.9$ is reached at roughly 8.5 dB post-processing SNR. Swerling III shifts this point to about 13.0 dB, and Swerling I, to about 17.0 dB. The ordering tracks the near-zero tail weight of each density: Swerling I places more probability at very low instantaneous RCS and demands a correspondingly larger mean margin. A deterministic link budget that ignores this spread will over-predict detection range for short-range perimeter defense.

**Figure 4:**
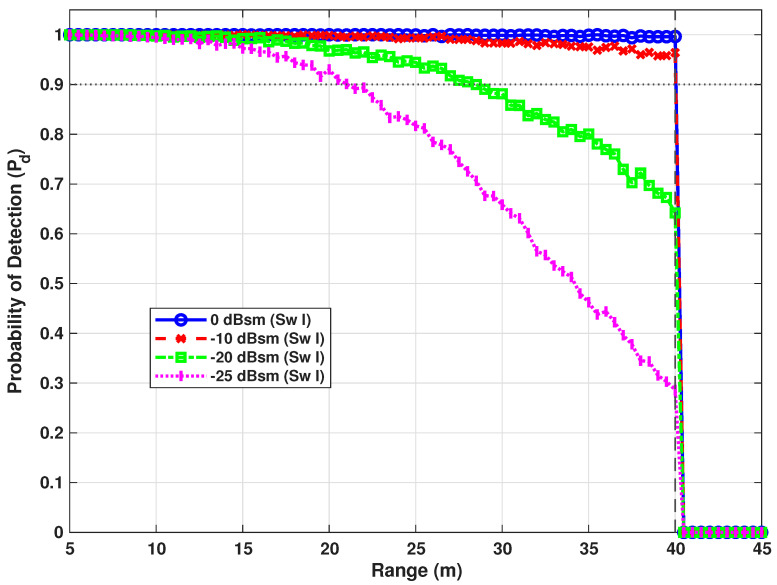
$P_{d}$ versus range, Swerling I ($P_{\mathrm{fa}}=10^{-6}$, 2000 trials). Dashed: 40 m limit.

Translating to range under Swerling I (Figure 4), the 0 and −10 dBsm classes hold $P_{d}$ > 0.9 across the full 40 m window. The picture changes sharply for lower cross-sections: $P_{d}\approx 0.9$ retreats to roughly 28 m at −20 dBsm and 21 m at −25 dBsm. Nano-UAV detectability is therefore margin-limited well inside the instrumented range. In a cluttered field deployment, adaptive thresholding and spatial filtering would partially offset this shortfall [12]. The trends are consistent with RCS data at comparable frequencies [3,16].

**Figure 5:**
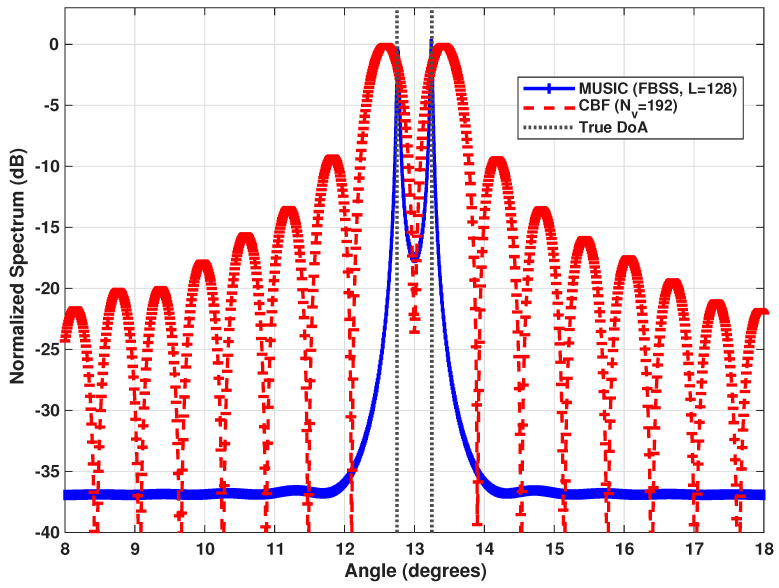
MUSIC and CBF spectra for two targets at 12.75° and 13.25° (20 dB SNR, *K* = 2, far-field model).

**Figure 6:**
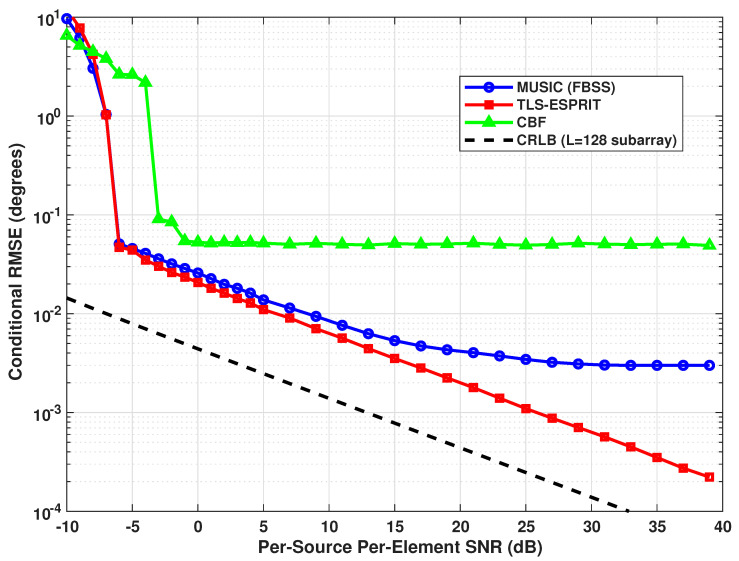
Conditional RMSE versus SNR (2000 trials, *K* = 2, far-field model). Off-grid targets at 12.503° and 13.503°.

**Figure 7:**
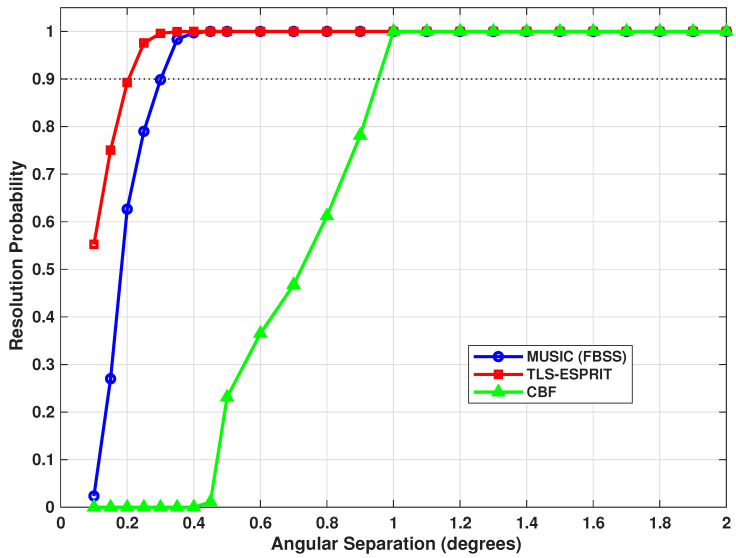
Resolution probability versus angular separation (15 dB SNR, *K* = 2, far-field model). Criterion: each estimate within max(Δθ/3, 0.05°) of its true angle and the pair separated by >Δθ/2.

Figure 5, Figure 6 and Figure 7 serve as idealized benchmarks for the assumed virtual ULA. They rely on plane-wave steering, white Gaussian noise, known source number, and perfect TDM compensation. Because targets at 10–25 m sit inside the near field of the full aperture, and because block-to-block phase error can be severe when Doppler quantization is coarse, these curves intentionally separate estimator performance from front-end model mismatch.

Figure 5 places two targets at 12.75° and 13.25°, a 0.5° separation, at 20 dB SNR. MUSIC resolves both peaks to within 0.02°, while CBF produces the broader mainlobes inherent to a diffraction-limited beamformer, with a corresponding error of about 0.16°. For co-range, co-velocity targets like Targets 2 and 3 in Table 6, this distinction determines whether the array can return two credible angle estimates or only a blurred composite peak.

Figure 6 broadens the comparison to off-grid targets at 12.503° and 13.503° with 1.0° separation (2000 trials per point). TLS-ESPRIT attains the lowest conditional RMSE in the moderate-SNR region; MUSIC trails closely, and CBF lags by a wide margin. The roughly 13–14 dB gap between TLS-ESPRIT and the plotted CRLB (and the approximately 15 dB gap for MUSIC) cannot be attributed to estimator quality alone. Part of it is structural: setting $N_{\mathrm{eff}}=P$ treats overlapping FBSS subarrays as independent, inflating the bound. Part is algorithmic: off-grid bias, finite-snapshot subspace leakage, and smoothing loss all prevent practical estimators from closing the gap tightly. In practical terms, the gap implies that reaching a given angular accuracy requires a snapshot SNR roughly 13–15 dB above what the plotted bound alone would suggest. This additional SNR requirement is the hardest to afford for the low-RCS targets that are already margin-limited in detection.

Figure 7 reframes the RMSE comparison in operational terms: the probability of resolving two targets as distinct arrivals. A trial succeeds if the estimator returns *K* = 2 estimates, each within max(Δθ/3, 0.05°) of its respective true angle, and the pair is separated by more than Δθ/2. Under this criterion, the angular separation needed for $P_{\mathrm{res}}\geq 0.9$ is roughly 0.25° for TLS-ESPRIT, 0.35° for MUSIC, and 1.0° for CBF. These numbers depend on the specific resolution criterion, but their relative ordering is stable and directly relevant to short-range airspace monitoring, where several low-altitude objects can share similar range–velocity bins while subtending well under a degree.

Extrapolating the favorable separations of Figure 7 to the 10–40 m operating region would be premature. Near-field wavefront curvature, calibration residuals, and imperfect TDM compensation all widen the effective separation needed in practice. Beamspace, iterative-adaptive, and deep-unfolded DoA methods have been developed precisely for scenarios where these departures from the ideal model become significant [31,33,34]. This interpretation is supported by the near-field estimate in Equation (9), which shows that the aperture-edge phase error over the 10–40 m range exceeds or approaches the usual far-field tolerance, especially for the full virtual aperture and for the smoothed subarray at shorter ranges. Likewise, Equation (8) shows that worst-case bin-quantized TDM compensation alone would bias the estimated angle by about 1.8°, several times the Rayleigh beamwidth, so the sub-degree separations reported above additionally presume sub-bin-accurate velocity compensation.

### 4.2. Micro-Doppler Matched-SNR Separability

**Figure 8:**
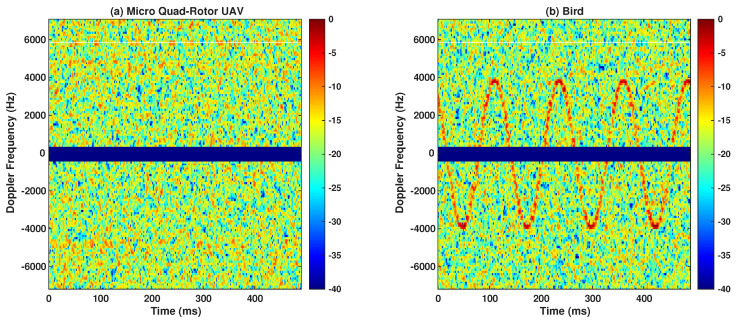
Pre-processed spectrograms at 15 dB ($T_{\mathrm{obs}}=0.5$ s): (**a**) micro-quadrotor; (**b**) bird.

Figure 8 illustrates the physical contrast driving the classification study. At a 15 dB total SNR over a 0.5 s window, the bird spectrogram captures only 4.0 wing-beat cycles, while every UAV class contributes at least 80 rotor cycles (Table 2). Operationally, this contrast cuts both ways. Even a fraction of the dwell captures many rotor cycles, so bird–UAV separation remains relatively robust. In contrast, the bird wing-beat modulation is sampled only about four times in 0.5 s, making the bird periodicity feature more sensitive to shortened operational dwells.

**Figure 9:**
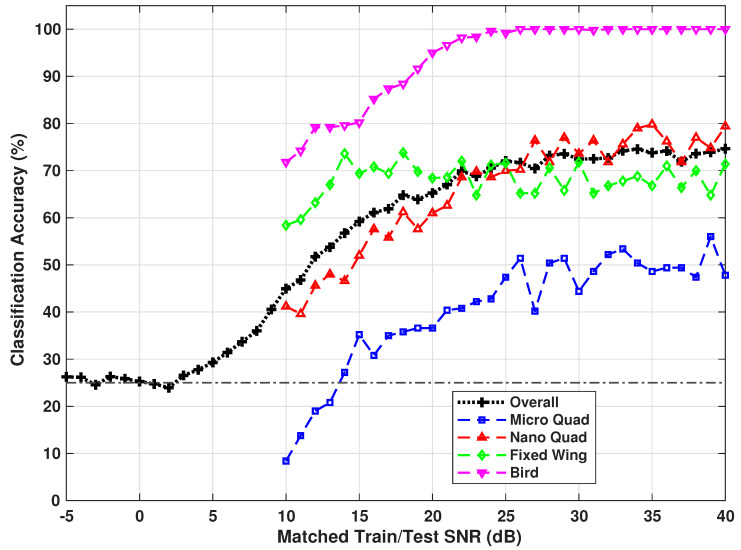
Classification accuracy versus matched SNR. Per-class curves for SNR ≥10 dB; dotted: 25% chance level.

Figure 9 plots matched-SNR classification with 300 training and 500 test samples per class at each SNR level. Overall four-class accuracy climbs from chance and plateaus near 72–75% above 25 dB. Birds separate far more readily, reaching about 95% at 20 dB and 99% at 25 dB; discrimination among the UAV subclasses remains comparatively poor. The corresponding UAV-only three-class accuracy levels off near 63%, confirming that the residual difficulty lies predominantly inside the UAV subset, not in bird–UAV rejection.

The confusion matrix in Table 7 pinpoints the source of error. Confusion clusters among the three UAV classes, with the heaviest exchange occurring between micro-quadrotor and fixed-wing targets. The physical explanation is overlap in their micro-Doppler feature values. Both classes yield comparable maximum bandwidths because their $f_{\mathrm{rot}}L_{b}$ products are close (100×0.12=12 m/s versus 80×0.20=16 m/s), and both share $N_{b}=2$ blades per rotor, generating similar harmonic structure. The number of rotors ($N_{r}=4$ versus $N_{r}=1$) does influence spectral entropy and harmonic count, but these differences are small compared with noise-induced variance. The nano-quadrotor separates more cleanly, consistent with its higher rotation rate and the resulting shift in the feature combination. A classifier with access to richer input (the full time–frequency image rather than four scalar features) could in principle leverage the distinct temporal patterns of single-propeller versus four-rotor modulation.

This plateau reflects the four-feature, nearest-centroid design, not a fundamental ceiling on radar-based classification. CNN and Transformer architectures operating on richer spectrogram representations have achieved stronger discrimination in comparable noise regimes [9,24,25]. Because training and testing use matched SNR and fixed class parameters, these curves are controlled separability experiments, not field-ready performance estimates.

Table 8 consolidates the principal results. Detection performs comfortably for the 0 and −10 dBsm classes across the full 40 m window, but very low-RCS targets hit margin limits well before the beat-frequency axis runs out. Angular estimation can be very sharp under the idealized far-field model, though that sharpness rests on assumptions known to be violated at the intended short ranges. Micro-Doppler classification separates birds from UAVs far more easily than it separates UAV subclasses from one another. This indicates that signature richness, not raw SNR, is the principal bottleneck of the present classifier.

The results in Table 8 should be interpreted as modular analytical and Monte Carlo benchmarks, not as complete deployment validation. Several assumptions were introduced intentionally to isolate the limiting mechanisms of the selected cascaded TDM-MIMO architecture. The detection analysis is noise-limited and uses a controlled Monte Carlo threshold, whereas a fielded radar would operate under clutter, multipath, moving vegetation, birds, vehicles, and possible mutual radar interference; thus, the relevant practical quantity is SINR rather than SNR, and any CFAR or interference-mitigation loss would reduce the detection range because received radar power follows the fourth-power range law. For example, an additional practical loss of *L* dB reduces the noise-limited range approximately as $R_{\mathrm{practical}}\approx R_{\mathrm{noise}}10^{-L/40}$, so losses of 3, 6, and 10 dB reduce the range to about 84%, 71%, and 56%, respectively. For instance, a standard CA-CFAR detector with 16–32 reference cells at $P_{\mathrm{fa}}=10^{-6}$ typically incurs about 1–2 dB of CFAR loss relative to an ideal fixed-threshold detector [35], corresponding to an additional range reduction of roughly 6–11% by the same fourth-power range scaling. The angular results are also optimistic because the DoA benchmark assumes far-field propagation, white Gaussian noise, known source number, ideal calibration, and perfect TDM compensation, even though near-field curvature, antenna-position errors, mutual coupling, inter-chip phase mismatch, temperature drift, and residual TDM phase error can distort the virtual-array manifold in the 10–40 m short-range window. Therefore, the MUSIC and ESPRIT resolution probabilities should be viewed as reference ceilings for the nominal aperture rather than direct field predictions. Similarly, the micro-Doppler classification results are controlled feature-space benchmarks: the synthetic data use fixed class parameters and broadside-only observation, while real targets would introduce aspect-angle variation, rotor-speed changes, maneuvering, nonstationary body Doppler, bird-species variability, clutter, and multipath. These factors are expected to broaden the feature distributions and reduce the accuracy of the four handcrafted features. Overall, the present framework provides a feasibility assessment of one cascaded 77 GHz TDM-MIMO parameterization and identifies where the margins are comfortable and where they are limited; experimental validation with measured 77 GHz drone and bird returns remains necessary before the configuration can be claimed as a field-ready counter-UAV sensing system.

It should also be emphasized that the present results are obtained through analytical modeling and Monte Carlo simulation; the described 12Tx × 16Rx, 77 GHz cascaded configuration has not been experimentally deployed in this study, and no claim of validated real-world performance is made. Nevertheless, the benchmark inputs are not arbitrary. The representative target cross-sections in Table 5 are based on reported millimeter-wave UAV and bird measurement studies [3,16], and the body-to-blade power ratio used in the micro-Doppler model follows measured drone-signature observations [16,20]. Therefore, the simulation inputs are empirically grounded, although the complete detection–DoA–classification processing chain has not yet been validated against raw measured radar returns. A direct comparison with external datasets would require compatible waveform parameters, array geometry, calibration conditions, target-aspect coverage, and synchronized range–Doppler–angle data, which are not available in the present study. Experimental validation with a cascaded 77 GHz front end and compatible measured drone and bird datasets remains a necessary future step before the proposed configuration can be claimed as a field-ready counter-UAV sensing system.

Based on the benchmark outcomes reported above, Table 9 positions the present work relative to representative prior studies.

## 5. Conclusions

The cascaded 12 Tx × 16 Rx front end provides comfortable detection margin for targets above −10 dBsm across the full 40 m window, but three factors limit its counter-UAV utility at lower cross-sections. First, Swerling I fluctuation imposes an 8.5 dB penalty relative to a deterministic target at $P_{d}\approx 0.9$, and the −20 and −25 dBsm classes fall below that threshold at roughly 28 and 21 m. Second, worst-case TDM residual phase on the twelfth transmit block reaches 11π/2 rad under two-fold zero-padding, far too large for coherent spatial processing, so the DoA benchmark was conducted under perfect compensation. In that idealized far-field setting, TLS-ESPRIT returned the lowest conditional RMSE, about 13–14 dB above the subarray CRLB, with $P_{\mathrm{res}}\geq 0.9$ at approximately 0.25° separation (versus 0.35° for MUSIC and 1.0° for CBF). Because the Fraunhofer distance exceeds the 10–40 m operating zone, these numbers are reference ceilings, not deployment predictions.

The micro-Doppler branch exposed a stark asymmetry. Birds reached 99% accuracy at 25 dB matched SNR, but four-class overall accuracy plateaued near 72–75% and UAV-only three-class accuracy near 63%. Confusion clusters between the micro-quadrotor and fixed-wing classes, whose $f_{\mathrm{rot}}L_{b}$ products and blade counts overlap. Richer representations (spectrogram images processed by lightweight CNNs or Transformer architectures) are the natural next step for intra-UAV discrimination.

These limitations also indicate the next steps needed before field use. The near-field phase error over the 10–40 m range reaches approximately 0.21π–0.84π rad for the full virtual aperture (Equation (9)), which is above the usual π/8 far-field tolerance. Therefore, the plane-wave DoA model should be replaced with spherical-wave steering, near-field focusing, or range-dependent calibration. In addition, bin-quantized TDM compensation can bias the angle estimate by about 1.8° (Equation (8)), which is several times the 0.53° Rayleigh beamwidth. Practical DoA processing therefore requires sub-bin Doppler interpolation, phase tracking, or joint velocity–angle estimation. The detection benchmark should also be extended to clutter and interference. Fielded operation is SINR-limited, and additional loss reduces the noise-limited range as $10^{-L/40}$; even CA-CFAR processing can add about 1–2 dB of loss. Thus, a deployment-oriented detection study should include clutter-aware thresholding and interference mitigation. For classification, the four handcrafted features plateau near 72–75% overall accuracy and 63% within the UAV subset. Moreover, a $45^{{}^{\circ}}$ aspect change can reduce the bandwidth feature by about 30%. Thus, the CNN- and Transformer-based classifiers identified above should be evaluated using measured or domain-randomized spectrograms with aspect, motion, and environmental variability. Finally, the complete detection–DoA–classification chain should be validated against measured 77 GHz drone and bird returns from a cascaded front end before any field-ready claim is made.

## Figures and Tables

**Table 1:** SNR convention summary.

Module	Symbol	Definition
Detection	${\mathrm{SNR}}_{\det,\mathrm{dB}}$	Post-combining detection SNR (dB)
DoA benchmark	SNR	Per-source per-element snapshot SNR
Micro-Doppler	SNR	Total (body+blade) power vs. noise

**Table 2:** Micro-Doppler parameters by target class.

Class	$N_{r}$	$N_{b}$	$L_{b}$ (m)	$f_{\mathrm{rot}}$ (Hz)	$f_{d,\max}$ (kHz)	Cycles/$T_{\mathrm{obs}}$ ^a^
Micro-quad	4	2	0.12	100	38.7	100
Nano-quad	4	2	0.05	200	32.3	200
Fixed-wing	1	2	0.20	80	51.6	80
Bird	2 ^b^	1	0.15	8	3.87	4.0

^a^ $N_{b}f_{\mathrm{rot}}\times T_{\mathrm{obs}}$; fundamental modulation cycles in 0.5 s. ^b^ Two “rotors” parameterize the two wings.

**Table 3:** System parameters.

Parameter	Symbol	Value
Carrier frequency	$f_{c}$	77 GHz
Wavelength	λ	3.896 mm
Tx/Rx antennas	$N_{\mathrm{Tx}}$/$N_{\mathrm{Rx}}$	12/16
Virtual-array elements	$N_{v}$	192
Chirp bandwidth	*B*	960 MHz
ADC sampling rate	$f_{s}$	4 MHz
Samples per chirp	$N_{s}$	256
Chirps per Tx block (detection)	$N_{c}$	128
*μ*D observation time	$T_{\mathrm{obs}}$	0.5 s (7143 chirps)
Chirp repetition interval	$T_{\mathrm{PRI}}$	70 *μ*s
Range resolution	$\delta_{R}$	0.156 m
Max. instrumented range	$R_{\max}$	40 m
Transmit power per channel	$P_{t}$	12 dBm
Antenna element gain	$G_{e}$	10 dBi
Receiver noise figure	*F*	14 dB
System losses	$L_{\mathrm{sys}}$	6 dB

**Table 4:** Reference link budget at $R_{0}=10$ m, σ=0 dBsm.

Parameter	Value	Unit
Transmit power, $P_{t}$	+12.0	dBm
Two-way antenna gain, $2G_{e}$	+20.0	dBi
Wavelength and spreading loss ^a^	−81.2	dB
Two-way path loss, $40\log_{10}\left(R_{0}\right)$	−40.0	dB
Target RCS	0.0	dBsm
System losses, $L_{\mathrm{sys}}$ ^b^	−6.0	dB
Received power	−95.2	dBm
Noise floor, $k_{B}T_{0}B_{n}$	−132.0	dBm
Noise figure, *F*	+14.0	dB
Noise power	−118.0	dBm
Single-chirp, single-bin SNR	+22.8	dB
Doppler integration gain, $10\log_{10}\left(N_{c}\right)$	+21.1	dB
Per-channel SNR (range–Doppler)	+43.9	dB
Non-coherent combining gain (approx.)	+11.4	dB
Detection SNR (approx.)	+55.4	dB

^a^ $10\log_{10}\left(\lambda^{2}/{\left(4\pi \right)}^{3}\right)=-81.2$ dB. ^b^ Atmospheric attenuation ≈0.02 dB, Hann ENBW loss ≈3.6 dB, and implementation margin ≈2.4 dB.

**Table 5:** Representative target RCS at 77 GHz (illustrative).

Target Class	Mean RCS (dBsm)	Model	Example
Large multirotor	−5 to 0	Swerling I	DJI Matrice
Micro-quadrotor	−15 to −10	Swerling I	DJI Mini
Nano-UAV	−25 to −20	Swerling I	Crazyflie
Fixed-wing mini	−20 to −10	Swerling I	Parrot Disco
Bird (avian)	−20 to −10	Swerling I	Pigeon

Ranges from [3,16]; order-of-magnitude values.

**Table 6:** Multi-target simulation scenario.

#	Class	*R* (m)	*v* (m/s)	*θ* (°)	RCS (dBsm)
1	Micro-UAV	10	1.1	8	−10
2	Micro-UAV	15	0.8	12	−10
3	Nano-UAV	15	0.8	18	−22
4	Fixed-wing	25	−0.5	−25	−8
5	Bird	20	0.3	5	−15

**Table 7:** Confusion matrix (%) at matched SNR = 25 dB.

True∖Pred.	Micro	Nano	Fixed	Bird
Micro-quadrotor	47	18	35	0
Nano-quadrotor	27	70	3	0
Fixed-wing	25	4	72	0
Bird	0	1	0	99

**Table 8:** Summary of key performance metrics.

Metric	Value
Maximum instrumented range	40 m
Detection SNR at 10 m, 0 dBsm	55.4 dB
$P_{d}\approx 0.9$ (SW0/SW III/SW I)	8.5/13.0/17.0 dB
$P_{d}\approx 0.9$ range (−20/−25 dBsm, SW I)	28/21 m
TLS-ESPRIT–CRLB gap (mod. SNR) ^†^	∼13–14 dB
MUSIC–CRLB gap (mod. SNR) ^†^	∼15 dB
$P_{\mathrm{res}}\geq 0.9$ (TLS-E/MUSIC/CBF) ^†‡^	∼0.25°/∼0.35°/∼1.0°
Worst-case TDM phase error (i=12, $Z_{D}=2$)	11π/2 rad
Four-class overall accuracy plateau	∼72–75%
UAV-only three-class accuracy plateau	∼63%
Bird accuracy at 25 dB matched SNR	99%
Micro-Doppler dwell	0.5 s (7143 chirps)

^†^ Idealized far-field benchmark. ^‡^ Criterion-dependent; approximate relative comparisons.

**Table 9:** Positioning of this work relative to representative prior studies. A checkmark (✓) indicates that the function is addressed; an en dash (–) indicates that it is not addressed; RCS denotes radar-cross-section measurements rather than an explicit detection benchmark.

Studies	Det.	DoA	*μ*D cls.	Evaluation Basis
UAV/bird measurement campaigns [3,16,17]	RCS	–	✓	Measured signatures; no joint system benchmark.
Handcrafted *μ*D features [19,20]	–	–	✓	Signal model/measured data.
Learned *μ*D/RD classifiers [9,21,24,25]	–	–	✓	Measured data + CNN/RNN.
Cascaded TDM-MIMO processing [13]	✓	✓	–	Processing-chain tutorial.
mmWave DoA algorithms [31,32,33,34]	–	✓	–	Algorithm-level studies.
**This work**	✓	✓	✓	Unified analytical/Monte Carlo benchmark of one fixed 12Tx × 16Rx, 77 GHz cascade, with quantified near-field and TDM phase-error limits.

## Data Availability

No measured experimental dataset was generated or analyzed in this study. The reported results are based on analytical modeling and Monte Carlo simulations; the main simulation settings and benchmark parameters are provided in the article.
